# Factors Affecting Migration Intentions of Polish Physiotherapists and Students of Physiotherapy—A Cross-Sectional Study

**DOI:** 10.3390/ijerph192114556

**Published:** 2022-11-06

**Authors:** Daria Kostrzewa, Joanna Bonior, Maciej Polak, Alicja Domagała

**Affiliations:** 1 Institute of Public Health, Faculty of Health Sciences, Jagiellonian University Medical College, 31-066 Krakow, Poland; 2 Department of Medical Physiology, Chair of Biomedical Sciences, Institute of Physiotherapy, Faculty of Health Sciences, Jagiellonian University Medical College, 31-126 Krakow, Poland

**Keywords:** migrations, migration of medical staff, physiotherapists, students of physiotherapy, factors driving migration

## Abstract

The phenomenon of professional migrations in the healthcare sector may exacerbate the problem of health workforce shortages. The scale of migration of medical personnel in Poland is estimated mainly on the certificates issued by the regional chambers confirming qualifications that grant the legal right to practice in other EU countries. Migrations concern also physiotherapists, who are the third largest group of health professionals. However, the problem of this phenomenon has not been assessed, and there is a lack of research in this area. The aim of the study was to compare the intention of migration among practicing physiotherapists and students in the last two years of master’s studies in physiotherapy, as well as to identify the factors affecting their intentions to migrate. The study covered practicing physiotherapists and students in the last two years of master’s studies in the field of physiotherapy in Poland. A total of 236 respondents took part in the study, including 119 physiotherapists and 117 students of physiotherapy. The tool used for the study was an online questionnaire. The scale of the intention to migrate was estimated at 45.3% among students and 47.1% in the group of practicing physiotherapists. The most frequently indicated destination countries for the migration of physiotherapy students and practicing physiotherapists were Germany, Norway, Switzerland, France and the United Kingdom. In both studied groups, the pull factors with the greatest impact on the intention to migrate were the possibility of obtaining higher earnings and working in better infrastructural conditions. In turn, the most important push factors turned out to be the low prestige of the profession in Poland, limited prospects for professional advancement and the stressful work environment. The respondents most often indicated separation from loved ones and poor command of foreign languages as significant barriers to professional migration. Both students of physiotherapy and practicing physiotherapists show great interest in the intention of professional migration, and the decisive determinant is economic factors.

## 1. Introduction

Health workforce migration is understood as a natural phenomenon that is caused by the development of societies and can be perceived as a global health issue of our time [1]. In 2019, the number of international migrants was estimated to be almost 272 million globally. It was about 3.5% of the world’s population, and it was 119 million more than in 1990. Nearly two-thirds of international migrants are labor migrants [2]. In recent years, the number of migrant physicians and nurses working in OECD countries has increased by 60% [3]. For example, in the United States, the United Kingdom, Australia and Canada, foreign nurses and doctors already account for 72% and 69% of all healthcare workers [4]. In Luxembourg, Sweden, Ireland, Switzerland or Malta, reliance on foreign medical doctors reported exceeded 20% [5].

The mobility of medical personnel has a twofold impact on the healthcare system’s function. Seemingly small outflows of medical staff do not cause significant systemic changes. International migration of medical personnel may contribute to the improvement in the functioning of health systems and to the increase in the provided services, presuming that the mass emigration of specialists does not apply to countries with low levels of economic development [6,7]. The above ethical aspect was highlighted in the 2010 WHO Global Code of Practice on the International Recruitment of Health Personnel [8]. Excessive emigration of medical specialists may lead to the failure of the health care system of the source country and an excessive burden of obligations on employees remaining in the system, which consequently may result in their further loss and a deepening crisis.

Migrations of healthcare workers are unpredictable, characterized by a fluctuation in mobility trends. In recent decades, countries such as Ireland and Spain have gone from being exporters of health professionals in the 1990s to being importers around the 2000s, to then again experiencing outflows of healthcare workers since around 2010 [5]. The constantly changing nature of mobility is a result of the multitude of factors influencing mobility. Factors affecting the decision to migrate include individual motivations, working conditions, economic circumstances in the home and destination country, legal frameworks and policy instruments. As a result of the migration of educated medical staff, the source country also incurs losses resulting from the loss of capital invested in the education process of future employees [9,10]. It shows a need to develop the tools to track the flows of healthcare professionals because no country can disregard health workers’ mobility or consider itself “safe” [5].

The topic of migration of healthcare workers plays an important role in political debates. Despite the general awareness of this global phenomenon, still, no coherent system of monitoring employee flows has been developed. In Poland, knowledge about the scale of migration is limited to the estimated data and concerns merely healthcare workers with medical chambers, which keep records of obtained certificates regarding the recognition of professional qualifications outside the country. The remaining statistical data comes from periodically conducted scientific research [6,11]. The scale of migration among doctors and nurses in Poland is estimated at about 7–9% [6,12]. The migration trend was significantly influenced by Poland’s accession to the European Union in May 2004, which opened new economic opportunities and guaranteed the free movement of people [13,14].

Proper identification of the main types of migration, as well as the factors influencing these decisions, is a key stage enabling effective measures to be taken to counteract the negative effects of this phenomenon. The basic analysis of the reasons prompting medical personnel to migrate is based on a two-factor model, which identifies push factors, which encourage people to leave their current place of residence, and pull factors, which trigger the desire to come to a new place [6,7]. Most of these factors work by contrast [15]. The main factor, both attracting and pushing out, is the amount of received remuneration and the resulting differences in income [6,7]. Moreover, decisions on migration are influenced by issues related to the infrastructural conditions in the workplace, administrative duties, prospects for career advancement or access to professional training [9,11,12]. Research conducted among medical workers shows that important factors that encourage migration include the prestige of the profession, opportunities for professional development and training, or family reasons, which sometimes necessitate a change of country of residence [6,12].

Increasing globalization and growth in demand for health services (in conjunction with the growing needs of an aging society) contribute to an increase in the emigration of qualified medical personnel, which also applies to professionals working as physiotherapists [15,16].

Physiotherapists are the third largest group of medical workers in the Polish healthcare system. According to the data provided by the National Chamber of Physiotherapists (KIF), the number of professionally active physiotherapists in 2020 was 66,250 [17]. There are about 17 physiotherapists per 10,000 inhabitants (about 600 inhabitants/per 1 physiotherapist), and approximately 73% of physiotherapists are women [18]. The average number of jobs is 1.4, and the average number of working hours is 145 h/month [19]. It should be emphasized that, compared to doctors and nurses, this is the group with the lowest average age; in 2020, it was 36.9 years, for doctors; it was almost 50 years; and for nurses, 52.2 years [17].

Due to the progressive aging of society, physiotherapists play an important role in the therapeutic process of patients requiring physiotherapeutic care or rehabilitation. Recent years have abounded in a number of changes in the physiotherapists’ education cycle and the rules of practicing the profession of a physiotherapist. In 2015, the government in Poland adopted a law on the profession of a physiotherapist, under which education in the field of physiotherapy is conducted in the form of five-year uniform master’s studies [20].

All practicing physiotherapists are obliged to belong to the National Chamber of Physiotherapists (KIF), which is the professional self-government of physiotherapists. It was established in 2015 to supervise the practice of the profession of a physiotherapist, represent members or work to improve their professional qualifications [20]. Therefore, both the position of physiotherapists in the healthcare system in Poland and their competencies regarding the provision of health services in the universal healthcare system have significantly improved [18].

According to the data reported by the Ministry of Family and Social Policy in the document entitled “Professions Barometer 2022”, the profession of a physiotherapist is one of the most scarce professions in Poland [21]. Therefore, it is necessary to investigate and improve the working conditions of people active in this profession and to monitor emigration intentions.

In the context of the migration of health professionals, including physiotherapists, national and EU legal provisions have great importance and regulate the possibility of taking up employment in the profession outside the country of citizenship. The profession of a physiotherapist is included in the list of regulated professions, for which it is necessary to have specific professional qualifications. Something that distinguishes the migration of medical staff from the migration of other occupations is the need for migrants to present appropriate certificates, which must be documented if an individual wants to work in their profession [13,22]. In Poland, the competent authority responsible for issuing certificates for physiotherapists is the National Chamber of Physiotherapists (KIF). According to the information obtained from KIF, from 2017 to September 2022, the National Chamber of Physiotherapists, at the request of Polish physiotherapists, issued 1493 certificates confirming the right to practice in another European Union country [23].

It needs to be highlighted that physiotherapists are the third most migrant group among regulated professions in Europe [22]. Before starting work in another European Union country, physiotherapists must apply for official recognition of qualifications in the destination country [13]. According to the current data published in the Regulated Profession Database, since Poland’s EU accession, 2920 physiotherapists who acquired their diploma in Poland confirmed their qualifications for the purpose of permanent establishment, as provided by EU Member States and EEFA countries (based on EU Directive 2005/36/WE) [22].

In general, the public discussion on the migration of healthcare professionals focuses on doctors and nurses. Research conducted in this area also tends to concern these two professional groups. In the context of the needs of the labor market, it is undoubtedly necessary to monitor this phenomenon in regard to physiotherapists. Therefore, it is important not only to determine the scale of intentions among working physiotherapists, but also to monitor migration plans among physiotherapy students, whose outflow from the labor market may significantly affect the efficiency of the health care system.

The main aim of the study was to compare the intention of professional migration among practicing physiotherapists and students in the last two years of master’s studies in the field of physiotherapy, as well as to explore the factors influencing their decisions to migrate.

## 2. Materials and Methods

### 2.1. Data Collection

A quantitative, cross-sectional survey of practicing physiotherapists and physiotherapy students in Poland was conducted between 10 January and 28 February 2022. The data were gathered via an online survey. The questionnaires were created by the authors and prepared using the Google Forms application. The link to the survey was published in seven nationwide social groups on Facebook that connect practicing physiotherapists and physiotherapy students. Participation in the study was voluntary. Participants were informed about the purpose of the study, their scientific and academic nature, anonymity and confidentiality.

### 2.2. Study Participants

The study covered practicing physiotherapists and students in the last two years of master’s studies in physiotherapy from different geographic areas in Poland. The survey was addressed to respondents from all over the country. The group of practicing physiotherapists included people from almost all Polish voivodeships (15 out of 16), while the group of students included people from 9 out of 16 voivodeships. At the time of the survey, the surveyed students attended 13 universities in Poland. The total number of completed questionnaires was 236. In the data analysis, we included 119 questionnaires from practicing physiotherapists and 117 questionnaires from students.

### 2.3. Ethical Procedures

The study was conducted anonymously on a sample of volunteers in accordance with the guidelines of the Code of Ethics for Researchers [24] and the Helsinki Declaration prepared by the World Medical Association [25].

### 2.4. Study Design

The study was conducted using a proprietary questionnaire, which was developed in two variants. The first variant was intended for a group of practicing physiotherapists and consisted of 22 questions. The second version was addressed to students of physiotherapy and contained 11 questions. The first part of both variants consisted of closed questions about sociodemographic characteristics. The second part included questions covering information on previous migrations, future migration intentions or the type of planned migration. The basic question regarding migration plans in the case of students was: “*Do you plan to migrate after graduation and obtain the right to practice a profession abroad?*”, and in the case of practicing physiotherapists: “*Are you currently considering practicing your profession abroad?*” The relation to the individual factors leading to potential migration was determined using a 5-item scale, where 1 meant no impact, and 5 meant a very large impact.

The release of the questionnaire was pre-tested by a pilot study, involving two physiotherapy students and two practicing physiotherapists. The purpose of the pilot study was to verify the prepared questions in terms of the adequacy and clarity of the questions formulated.

### 2.5. Statistical Analysis

Variables are expressed as numbers (*n*) and percentages (%) or as a median (Me) and interquartile range (Q_1_–Q_3_), as appropriate. The Shapiro–Wilk test was used to assess conformity with a normal distribution. The assessment of the relationship between selected characteristics of the studied groups and the intention to migrate was performed using the X^2^ test or the Mann–Whitney U test, as appropriate. Statistical analyses were performed using the Statistica 13.3 program (TIBCO Software Inc., Palo Alto, CA, USA). Values of *p* < 0.05 were accepted as statistically significant.

## 3. Results

A total of 236 questionnaires were analyzed. The students’ median age was 23 years, and the practicing physiotherapists’ median age was 30 years. The group of students included 96 women and 21 men, while the group of practicing physiotherapists included 72 women and 47 men. In the group of physiotherapy students were 58 respondents (49.6%) in the fourth year of studies and 59 respondents in the fifth year of studies (50.4%). The dominant part of the group was students pursuing full-time studies (88%). The group of practicing physiotherapists was dominated by respondents with higher education (99.2%). The majority of practicing physiotherapists (86 people (72.3%)) were employed in one healthcare facility providing medical services. A total of 33 practicing physiotherapists (27.7%) declared employment in two or more workplaces. More than half of the practicing physiotherapists were employed on the basis of a full-time or part-time employment contract (63 people (53%)). Almost 1/3 of the respondents also reported an employment relationship on the basis of a civil law contract, and nearly 32% of physiotherapists performed individual economic activity (Table 1).

### 3.1. Migration Plans

**Figure 1:**
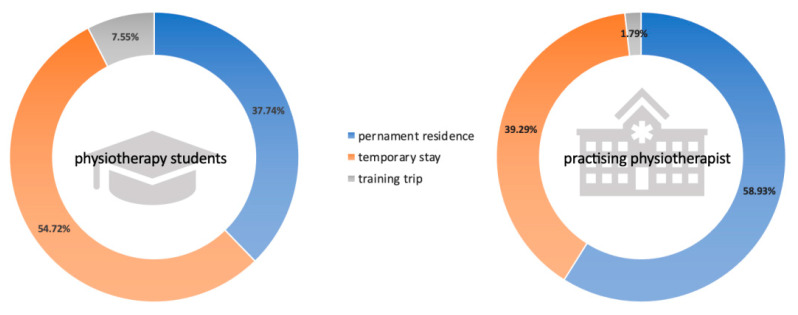
Comparison of the purpose of migration of physiotherapy students and practicing physiotherapists.

Intention to migrate was declared by a total of 109 (46.2%) respondents. The frequency of willingness to migrate did not differ between students and practicing physiotherapists (45.3% vs. 47.1%, *p* = 0.79). Figure 1 shows a comparison of the types of migration between students and practicing physiotherapists. Students declared the purpose of migration as a training trip 4 times more often and almost 2 times less often for permanent residence than practicing physiotherapists; however, these relationships were on the border of statistical significance (*p* = 0.053).

In the group of students no statistically significant differences were observed between sex, marital status and year of study, and the intention to migrate (see Table S1 in Supplementary Materials).

In the group of practicing physiotherapists, a statistically significant association was demonstrated between having children and the intention to migrate. Participants with children less frequently declared their intention to migrate. The type of employment in the workplace, which is the primary source of income, was also significantly associated with the intention to migrate among practicing physiotherapists. Physiotherapists employed on the basis of an employment contract or running their own business, compared to people employed on the basis of civil law contracts, also less frequently declared their intention to migrate. People who were in a relationship (formal or informal) were also less likely to intend to migrate than single people. There were no statistically significant differences between age, length of service, weekly workload, number of employment entities and the intention to migrate among physiotherapists (Table 2).

### 3.2. Migration Factors

**Figure 2:**
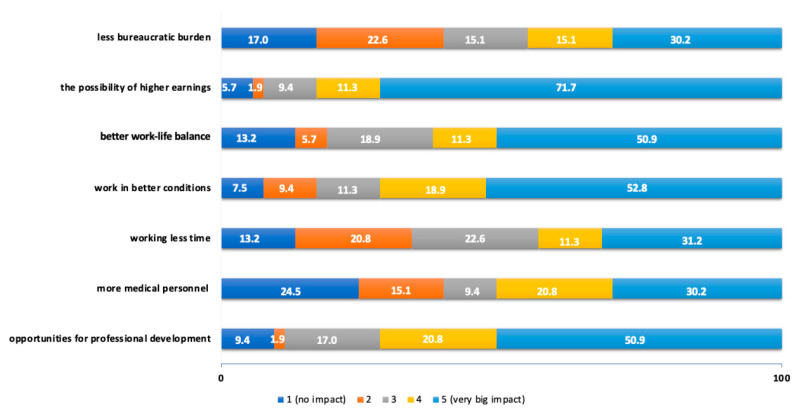
Assessment of the influence of pull factors according to students of physiotherapy.

More than 50% of physiotherapy students indicated the 4 and 5 values when asked to assess the following pull factors: the possibility of higher earnings (83%), working in better conditions (71.7%), opportunities for professional development (71.7%) or better work–life balance (62.2%) (Figure 2).

**Figure 3:**
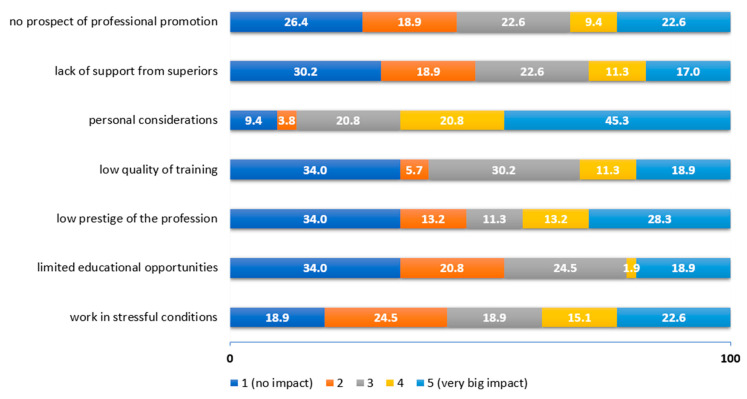
Assessment of the influence of push factors according to students of physiotherapy.

On the other hand, the most important pushing factors for physiotherapy students were personal considerations (66.1%), low prestige of the profession in Poland (41.5%), work in stressful conditions (37.7%) and no prospects of professional promotion (32%) (Figure 3).

**Figure 4:**
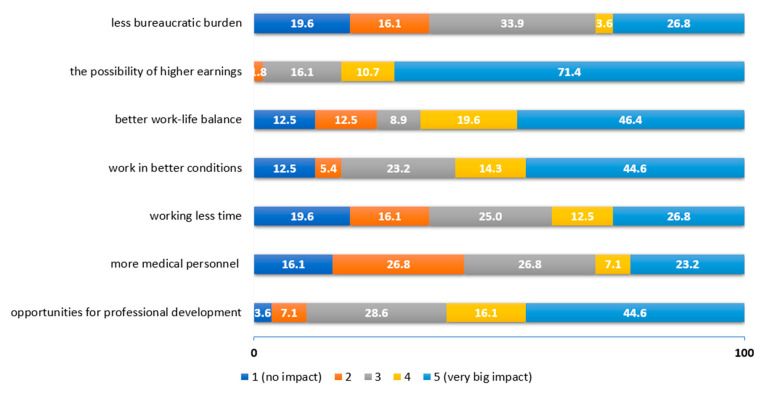
Assessment of the influence of pull factors according to practicing physiotherapists.

The most important pull factors for practicing physiotherapists are the possibility of higher earnings (82.1%), better work–life balance (66%), greater opportunities for professional development in other countries (60.7%) and working in better infrastructural conditions (58.9%) (Figure 4).

**Figure 5:**
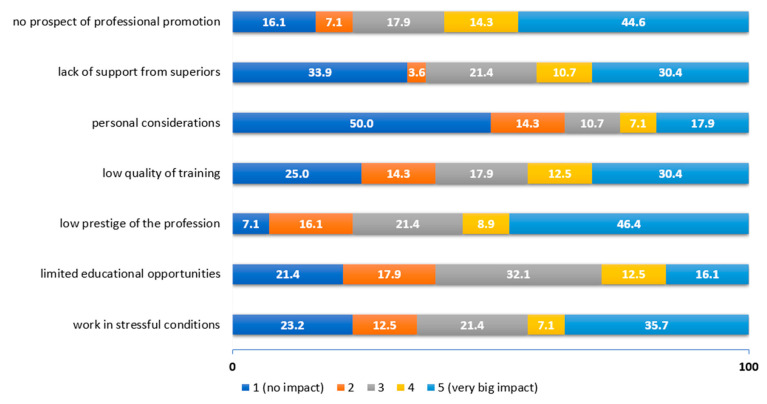
Assessment of the influence of push factors according to practicing physiotherapists.

On the other hand, the most important push factors were the low prestige of the profession in Poland (55.3%), no prospect of professional promotion (58.9%) and work in stressful conditions (42.8%) (Figure 5).

### 3.3. Destination Countries for Migration

The most frequently declared countries of potential migration of physiotherapy students were Germany (32.1%), Norway (26.4%), France (24.5%), Switzerland (24.5%) and the United Kingdom (24.5%). In turn, the most common destination countries indicated by practicing physiotherapists were: Germany (42.9%), Switzerland (28.6%), Norway (25.0%), France (23.2%) and the United Kingdom (17.9%).

### 3.4. Barriers to Emigration

The majority of physiotherapy students (62.7%) indicated “differences in education” as a migration barrier. Physiotherapy students also indicated leaving family (60.8%) and language barrier (58.8%) as significant barriers to migration. Almost 55.4% of practicing physiotherapists indicated language as a migration barrier, while 46.4% and 37.5% indicated leaving family and fear of a new work environment.

## 4. Discussion

In our study, the scale of the intention to migrate was estimated at a high level, i.e., 45.3% among students and 47.1% in the group of practicing physiotherapists.

Similar results were obtained in another study conducted among students of physiotherapy and paramedics at one of the Polish medical universities, where 44.6% of the respondents declared their willingness to work abroad [26]. In a study by Szostek et al., conducted on a group of nursing students at the Medical University of Warsaw, as many as 53% of respondents declared their intention to migrate [27].

The scale of declared emigration by practicing physiotherapists is clearly higher compared to other Polish medical professionals: the declared willingness to emigrate concerns approximately 34% of doctors [28] and 21.5% of nurses [29,30]. This may be due to the fact that physiotherapists are a group with a much lower average age compared to doctors and nurses. Studies from other countries also confirm that younger medical professionals declare a greater willingness to migrate compared to their older colleagues [6,31,32,33]. The current generation of young medical specialists, including physical therapists, values work–life balance highly; they expect not only better wages, but also better working conditions and flexibility of working hours.

Our results are in line with other European studies. The results of studies conducted among Croatian students of physiotherapy indicated that 50% of the surveyed students showed an intention to migrate [34]. According to the study conducted among Hungarian physiotherapists, the results were similar: 50.6% of the respondents (*n* = 109) confirmed their intentions to leave the profession. The most common reasons are unfavorable financial and moral recognition and the lack of possibilities for career development [35].

In both examined subgroups of our study, the pull factors with the greatest impact on the intention to migrate were the possibility of higher earnings, working in better infrastructural conditions, the chance for work–life balance and opportunities for professional development. In turn, the most important push factors turned out to be the low prestige of the profession in Poland, no prospects for professional promotion and work in stressful conditions. The obtained results coincide with the results of other studies conducted among medical students, for whom the main motivators for going abroad were higher earnings, better working conditions and development prospects [26,27,36]. Similar results are reported in studies conducted among medical students in other European countries [32,33,37,38].

The reasons for migration indicated in the studies conducted among Polish doctors or nurses also correspond to the factors dominating among the surveyed physiotherapists [28,29]. The results of the research confirm that the low level of remuneration of medical staff in Poland is one of the most serious problems constituting the health workforce shortages. For this reason, Poland is perceived as an unattractive destination from the point of view of foreign healthcare workers, which has so far been a characteristic feature of countries with a lower economic status. The current situation, related to under-financing and relatively low expenditure on health care compared to other European Union countries, therefore does not favor the phenomenon of brain exchange, i.e., a two-way flow of specialists between countries, which may partially increase employment rates in the national health system [12].

According to the results of an international study conducted by Cahalin et al., the level of physiotherapists’ remuneration varies significantly among countries (40 countries were included in the study), but with greater experience professionals consistently exceed the average salary in almost all included countries [15]. Moreover, despite the earning potentials being very different, in over 50% of the analyzed countries, an entry-level salary exceeded the average worker’s salary and the salary of professionals with 5 or more years of experience exceeded the average worker’s salary in all countries [15]. The salary level of physiotherapists in Poland is unsatisfactory and much lower than the national average [19]. In 2019, the average monthly salary in Poland was PLN 3680 net, and the average salary of a physiotherapist employed under an employment contract was only PLN 2158 net [19]. Currently, salary increases are being implemented as a result of the amendment to the act on the method of determining the minimum base salary of some employees of healthcare entities [39]. Under this act, from July 2022, healthcare entities increased the salary of medical workers, depending on what has been established in the so-called “labor factors” for a given group of employees. The average monthly gross wages and salaries in the national economy of Poland in 2021 amounted to PLN 5662.53 [40]. In accordance with the provisions set out in the above-mentioned act, with coefficients, depending on the level of education and specializations, three variants of remuneration for physiotherapists were established [40]. However, it is too early to assess whether the scale of the increase in remuneration contributed to the increase in the satisfaction of physiotherapists.

Significant barriers to professional migration, both among the surveyed students and in the group of practicing physiotherapists, turned out to be leaving family and language barriers. The necessity to leave the family was also the greatest difficulty for doctors and nurses [28,29,30].

Statistical analysis of the group of practicing physiotherapists showed statistical significance between having children, the type of employment and the intention to migrate. Childless physiotherapists and those employed on the basis of civil law contracts more often declared their intention to go abroad. A similar relationship was noticed in studies conducted among physicians [28].

The most frequently indicated target countries for the migration of physiotherapy students and practicing physiotherapists were Germany, Norway, Switzerland, France and the United Kingdom. The respondents preferred countries that offer a higher standard of living and the possibility of improving the financial situation, which was also noticeable in other publications concerning Polish doctors, nurses or medical students [6,12,28,29,30].

### 4.1. Implications of the Study and Recommendations for Further Research

The results of the study show a great interest in professional migration, both among students in the last two years of studies in physiotherapy, as well as among practicing physiotherapists. The results of research conducted in this area among other professional groups also confirm the importance of this phenomenon. There is an undeniable need to implement a system for monitoring the flow of healthcare workers, as well as to take measures at the government level to retain medical staff in the Polish healthcare system. In addition, the desired process would be to increase the financing of the healthcare sector and to implement a real increase in salaries. It could make Poland a more attractive country for foreign medical personnel, which would allow reducing health workforce shortages. Due to the very important and constantly growing role of physiotherapists in the health system, measures should be taken to improve their working conditions and professional satisfaction. This requires monitoring of factors that influence the level of satisfaction and thus plays a role in decisions about possible professional migration.

### 4.2. Strengths and Limitations

To our knowledge, the presented study is the first study in Poland to assess the scale of declared emigration both in the group of physiotherapists and students in the last years of physiotherapy studies.

The study has some limitations. The first is a relatively small number of respondents, both among students of physiotherapy and working physiotherapists. However, the described study can be treated as a pilot and a starting point for a large nationwide study on a large group of representatives of this profession.

## 5. Conclusions

The analysis of migration intentions among both physiotherapy students and practicing physiotherapists confirmed the high interest in migration. The key factors prompting professional migration in both surveyed groups were economic issues, work in better infrastructural conditions, opportunities for better work–life balance and opportunities for professional development. The identified factors influencing the decision to emigrate relate mainly to unsatisfactory working conditions. Considering the huge demand of the domestic labor market for the services of physiotherapists, urgent systemic measures should be taken to retain them in the Polish health system. Long-term strategy and comprehensive actions are needed to improve the working conditions and job satisfaction of Polish physiotherapists. Moreover, monitoring migration trends should be implemented. It is also necessary to conduct further in-depth research and analysis in this area.

## Figures and Tables

**Table 1:** Characteristics of the physiotherapy students and practicing physiotherapists.

Students of Physiotherapy (*n* = 117)		Practicing Physiotherapist (*n* = 119)	
Parameter		Parameter	
Sex, *n* (%)		Sex, *n* (%)	
woman	96 (82.1%)	woman	72 (60.5%)
man	21 (17.9%)	man	47 (39.5%)
Age, Me (Q_1_–Q_3_):	23 (23–24)	Age, Me (Q_1_–Q_3_):	30 (27–35)
Marital status, *n* (%)		Marital status, *n* (%)	
lonely person	72 (61.5%)	lonely person	49 (41.2%)
person in relationship	45 (38.5%)	person in relationship	70 (58.8%)
Year of study, *n* (%)		Education, *n*(%)	
4th year	58 (49.6%)	medium	1 (0.8%)
5th year	59 (50.4%)	bachelor’s degree	1 (0.8%)
Form of studies, *n* (%)		master’s degree	114 (95.8%)
full-time studies	103 (88.0%)	doctoral degree	3 (2.6%)
part-time studies	14 (12.0%)	Having children, *n* (%)	
		yes	34 (28.6%)
		no	85 (71.4%)
		Number of places of employment, *n* (%)	
		1 place	86 (72.3%)
		2 places or more	33 (27.7%)
		Employment basis, *n*(%)	
		employment contract (full-time)	59 (49.6%)
		civil law contract	24 (20.2%)
		economic activity	36 (30.2%)
		Work experience, year Me (Q_1_–Q_3_):	5 (3–10)
		Weekly workload, hour Me (Q_1_–Q_3_):	40 (35–45)

**Table 2:** Characteristics of practicing physiotherapists according to migration intentions.

Variable	Migration Plans				*p*
	No (*n* = 63)		Yes (*n* = 56)		
Sex, *n* (%)					
woman	39	54.2	33	45.8	0.740 ^A^
man	24	51.1	23	48.9	
Marital status, *n* (%)					
lonely person	20	40.8	29	59.2	0.026 ^A^
person in relationship	43	61.4	27	38.6	
Having children, *n* (%)					
no	39	45.9	46	54.1	0.015 ^A^
yes	24	70.6	10	29.4	
Type of employment, *n* (%)					
contract of employment	34	57.6	25	42.4	0.031 ^A^
civil law contract	7	29.2	17	70.8	
economic activity	22	61.1	14	38.9	
Number of places of employment, *n* (%)					
1 place	46	53.5	40	46.5	0.847 ^A^
2 places or more	17	51.5	16	48.5	
Age, Me (Q1–Q3)	30 (27–36)		29 (27–33)		0.207 ^B^
Work experience, yearsMe (Q1–Q3)	5 (3–10)		4 (3–7.5)		0.098 ^B^
Weekly workload, hoursMe (Q1–Q3)	38 (35–45)		40 (32–45)		0.603 ^B^

Data are presented as ^A^—*p*-value from X^2^ test, ^B^—*p*-value from U Mann–Whitney test.

## Data Availability

Not applicable.

## Supplementary Materials

The following supporting information can be downloaded at: https://www.mdpi.com/article/10.3390/ijerph192114556/s1, Table S1: Results of statistical analyses among students of physiotherapy.
