# Supplementary material for: Factors Affecting Migration Intentions of Polish Physiotherapists and Students of Physiotherapy—A Cross-Sectional Study
Source: Int J Environ Res Public Health. 2022 Nov 6;19(21):14556. doi: 10.3390/ijerph192114556 (PMC9658116; doi:10.3390/ijerph192114556)
Supplement: Supplementary file 1 [file ijerph-19-14556-s001.zip › ijerph-1997298-supplementary.pdf]

SUPPLEMENTARY MATERIALS

Table S1. Results of statistical analyzes among students of physiotherapy

**variable**	**migration plans**				**p**
	**no (n=64)**		**yes (n=53)**		
	n	%	n	%	
**Sex, n (%)**					
woman	56	58.3	40	41.7	0.092^{A}
man	8	38.1	13	61.9	
**Marital status, n (%)**					
lonely person	35	48.6	37	51.4	0.094^{A}
person in relationship	29	64.4	16	35.6	
**Year of study, n (%)**					
4th year	32	55.2	26	44.8	0.919^{A}
5th year	32	54,2	27	45.8	

Data are presented as A - p-value from X^2 test
